# Supplementary material for: Antibody evasion by the P.1 strain of SARS-CoV-2
Source: Cell. 2021 May 27;184(11):2939–2954.e9. doi: 10.1016/j.cell.2021.03.055 (PMC8008340; doi:10.1016/j.cell.2021.03.055)
Supplement: Document S1. Tables S1–S5 [file mmc1.pdf]

Cell, Volume *184*

Supplemental information

Antibody evasion by the P.1 strain of SARS-CoV-2

Wanwisa Dejnirattisai, Daming Zhou, Piyada Supasa, Chang Liu, Alexander J. Mentzer, Helen M. Ginn, Yuguang Zhao, Helen M.E. Duyvesteyn, Aekkachai Tuekprakhon, Rungtiwa Nutalai, Beibei Wang, César López-Camacho, Jose Slon-Campos, Thomas S. Walter, Donal Skelly, Sue Ann Costa Clemens, Felipe Gomes Naveca, Valdinete Nascimento, Fernanda Nascimento, Cristiano Fernandes da Costa, Paola Cristina Resende, Alex Pauvolid-Correa, Marilda M. Siqueira, Christina Dold, Robert Levin, Tao Dong, Andrew J. Pollard, Julian C. Knight, Derrick Crook, Teresa Lambe, Elizabeth Clutterbuck, Sagida Bibi, Amy Flaxman, Mustapha Bittaye, Sandra Belij-Rammerstorfer, Sarah C. Gilbert, Miles W. Carroll, Paul Klenerman, Eleanor Barnes, Susanna J. Dunachie, Neil G. Paterson, Mark A. Williams, David R. Hall, Ruben J.G. Hulswit, Thomas A. Bowden, Elizabeth E. Fry, Juthathip Mongkolsapaya, Jingshan Ren, David I. Stuart, and Gavin R. Screaton

Table S1 Data collection and refinement statistics of RBD complexes, related to X-ray data collection, structure determination and refinement, STAR Methods

**Structure**	**RBD-EY6A-222**	**K417N-RBD-EY6A-222**	**K417T-RBD-EY6A-222**	**B.1.1.7-RBD-EY6A-222**	**P.1-RBD-EY6A-222**	**B.1.351-RBD-EY6A-222**	**P.1-RBD-ACE2**
**PDB ID**	7NX6	7NX7	7NX8	7NX9	7NXB	7NXA	7NXC
**Data collection**							
Space group	$P2_12_12_1$	$P2_12_12_1$	$P2_12_12_1$	$P2_12_12_1$	$P2_12_12_1$	$P2_12_12_1$	$P4_12_12$
Cell dimensions							
a, b, c (Å)	54.4, 120.1, 211.7	54.7, 122.4, 213.7	54.8, 122.7, 213.9	54.3, 120.9, 210.2	54.7, 122.8, 212.3	54.2, 120.4, 211.6	103.5, 103.5, 225.9
a, b, g (°)	90, 90, 90	90, 90, 90	90, 90, 90	90, 90, 90	90, 90, 90	90, 90, 90	90, 90, 90
Resolution (Å)	120–2.25 (2.29–2.25)[a]	81–2.30 (2.30–2.34)	81–1.95 (1.98–1.95)	79–2.40 (2.44–2.40)	80–2.67 (2.72–2.67)	61–2.50 (2.54–2.50)	70–3.14 (3.27–3.14)
R_{merge}	0.438 (---)	0.245 (---)	0.120 (0.990)	0.257 (---)	0.365 (---)	0.401 (---)	0.500 (---)
R_{pim}	0.073 (0.767)	0.039 (0.367)	0.034 (0.972)	0.051 (0.547)	0.072 (1.176)	0.080 (1.086)	0.100 (1.087)
I/s(I)	4.3 (0.3)	7.7 (0.4)	12.0 (0.4)	7.7 (0.6)	7.0 (0.4)	4.6 (0.3)	5.3 (0.5)
$CC_{1/2}$	0.991 (0.320)	0.997 (0.664)	0.996 (0.354)	0.995 (0.584)	0.997 (0.344)	0.992 (0.360)	0.992 (0.306)
Completeness (%)	99.8 (95.8)	100 (99.7)	100 (99.6)	100 (98.5)	100 (97.7)	99.9 (94.2)	100 (98.1)
Redundancy	34.8 (17.3)	39.6 (36.0)	13.4 (13.2)	26.6 (25.8)	26.3 (27.5)	26.3 (27.1)	25.7 (27.1)
**Refinement**							
Resolution (Å)	106–2.25	81–2.30	81–1.95	79–2.40	80–2.67	61–2.50	70–3.14
No. reflections	61666/3310	6175/3233	100792/5255	52327/2727	39534/2103	45980/2393	20278/1904
R_{work} / R_{free}	0.207/0.248	0.208/0.239	0.223/0.246	0.200/0.229	0.213/0.251	0.205/0.253	0.226/0.278
No. atoms							
Protein	8014	8004	8017	8012	8025	8014	6405
Ligand/ion/water	386	373	389	345	182	192	70
B factors ($Å^2$)							
Protein	67	87	78	72	91	77	90
Ligand/ion/water	60	72	50	67	84	92	102
r.m.s. deviations							
Bond lengths (Å)	0.002	0.003	0.002	0.002	0.002	0.002	0.003
Bond angles (°)	0.5	0.6	0.5	0.5	0.5	0.5	0.6

[a]Values in parentheses are for highest-resolution shell.

Table S2 Monoclonal antibody neutralization and binding data, related to Figures 2 and 3

mAb	IC50 (ug/ml)				FRNT^{50} ratio			KD (nM)					Immunoglobulin gene usage		
	Victoria	B.1.1.7	B.1.351	P.1	B.1.1.7/ Viictoria	B.1.351/ Victoria	P.1/ Victoria	Native RBD	K417T	K417N	E484K	RBD P.1	IGHV	K/λ	IGLV
40	0.026 ± 0.007	0.035 ± 0.008	0.738 ± 0.311	0.153 ± 0.037	1.4	28.6	5.9	1.33±0.03	38.90±0.89	56.7±1.18	7.31±0.03	38.20±0.95	3-66	K	1-33 or 1D-33
55	0.095 ± 0.015	0.348 ± 0.044	0.127 ± 0.014	0.306 ± 0.046	3.7	1.3	3.2	2.66±0.05	4.60±0.12	4.21±0.09	3.82±0.02	11.10±0.37	1-58	K	3-20
58	0.041 ± 0.003	0.116 ± 0.029	0.136 ± 0.010	0.236 ± 0.075	2.8	3.3	5.7	1.46±0.04	1.83±0.02	2.75±0.03	0.97±0.04	2.84±0.03	3-9	λ	3-21
88	0.033 ± 0.001	0.058 ± 0.008	>10	>10	1.8	>307.6	>307.6	2.90±0.03	Knocked out	Knocked out	5.37±0.06	Knocked out	4-61	λ	1-36
132	0.048 ± 0.000	0.337 ± 0.048	>10	>10	7.0	>208.4	>208.4	2.93±0.10	1846±364	2495±1095	29.80±0.36	1338±452	4-34	λ	7-46
150	0.012 ± 0.000	0.139 ± 0.019	0.350 ± 0.010	0.040 ± 0.003	12.0	30.0	3.4	0.40±0.01	1.64±0.02	3.35±0.03	1.83±0.03	8.90±0.13	3-53	K	1-9
158	0.031 ± 0.004	0.254 ± 0.109	>10	>10	8.3	>327.5	>327.5	1.89±0.03	4.83±0.06	9.35±0.11	3.03±0.10	18.20±0.31	3-53	K	1-9
159	0.011 ± 0.000	0.061 ± 0.020	>10	1.434 ± 0.804	5.7	>928.4	133.2	N/A	N/A	N/A	N/A	N/A	3-30	K	3-20
165	0.034 ± 0.004	0.212 ± 0.004	0.054 ± 0.013	0.241 ± 0.030	6.3	1.6	7.2	2.15±0.03	2.62±0.06	3.29±0.05	3.49±0.03	7.10±0.15	1-58	K	3-20
170	0.025 ± 0.004	0.105 ± 0.050	>10	>10	4.2	>402.2	>402.2	5.23±0.02	6.24±0.04	6.95±0.05	Knocked out	463.4±25.4	5-51	K	2D-29
175	0.026 ± 0.000	0.575 ± 0.280	>10	3.881 ± 0.738	22.5	>391.5	151.9	1.36±0.01	3.17±0.02	8.60±0.04	10.8±0.07	68.30±0.50	3-53	K	1-33 or 1D-33
222	0.019 ± 0.000	0.014 ± 0.002	0.017 ± 0.005	0.008 ± 0.003	0.7	0.9	0.4	1.36±0.08	3.96±0.11	5.16±0.12	2.25±0.04	1.92±0.01	3-53	K	3-20
253	0.055 ± 0.008	0.126 ± 0.018	0.109 ± 0.055	0.137 ± 0.005	2.3	2.0	2.5	1.15±0.03	5.99±0.11	6.11±0.11	2.66±0.03	4.25±0.11	1-58	K	3-20
269	0.030 ± 0.000	>10	>10	>10	>337.5	>337.5	>337.5	0.76±0.02	6.69±0.04	8.88±0.04	1.29±0.03	Knocked out	3-53	K	1-9
278	0.014 ± 0.007	0.307 ± 0.149	0.160 ± 0.018	0.245 ± 0.042	22.5	11.7	17.9	4.16±0.03	5.21±0.03	6.37±0.03	6.95±0.27	16.20±0.17	1-18	K	1-39 or 1D-39
281	0.005 ± 0.001	0.012 ± 0.000	>10	>10	2.5	>2026.3	>2026.3	0.97±0.03	0.68±0.02	4.42±0.03	3.54±0.08	Knocked out	3-7	K	2-24
316	0.018 ± 0.007	0.024 ± 0.005	>10	>10	1.4	>563.6	>563.6	4.81±0.05	4.96±0.06	5.17±0.08	Knocked out	Knocked out	1-2	λ	2-8
318	0.029 ± 0.008	0.185 ± 0.037	0.019 ± 0.008	0.083 ± 0.032	6.3	0.7	2.8	4.43±0.04	4.96±0.04	5.35±0.04	6.05±0.02	9.30±0.03	1-58	K	3-20
384	0.004 ± 0.001	0.005 ± 0.002	>10	>10	1.1	>2398.4	>2398.4	1.19±0.02	1.30±0.03	1.80±0.03	1.75±0.04	Knocked out	3-11	K	1-27
398	0.091 ± 0.004	0.180 ± 0.001	>10	>10	2.0	>110.2	>110.2	4.63±0.04	10.40±0.10	13.60±0.24	Knocked out	Knocked out	3-66	λ	2-8
AZD1061	0.013 ± 0.003	0.012 ± 0.002	0.014 ± 0.002	0.007 ± 0.002	0.9	1.1	0.5	5.13±0.07	8.00±0.12	6.14±0.08	7.66±0.05	2.27±0.03	N/A	N/A	N/A
AZD8895	0.005 ± 0.001	0.011 ± 0.002	0.046 ± 0.031	0.046 ± 0.016	2.2	8.9	8.8	2.18±0.04	2.96±0.05	3.56±0.06	8.50±0.05	7.35±0.15	N/A	N/A	N/A
AZD7442	0.009 ± 0.000	0.007 ± 0.001	0.012 ± 0.001	0.006 ± 0.003	0.8	1.4	0.7	N/A	N/A	N/A	N/A	N/A	N/A	N/A	N/A
REGN10987	0.032 ± 0.007	0.028 ± 0.003	0.007 ± 0.001	0.013 ± 0.002	0.9	0.2	0.4	1.35±0.03	1.61±0.03	1.84±0.04	1.39±0.05	1.38±0.02	N/A	N/A	N/A
REGN10933	0.004 ± 0.002	0.014 ± 0.002	3.284 ± 2.014	6.177 ± 1.914	3.3	773.7	1455.2	0.97±0.02	1.99±0.02	1.70±0.01	1.92±0.02	308.7±10.0	N/A	N/A	N/A
ADG10	0.006 ± 0.000	0.010 ± 0.001	0.011 ± 0.001	0.003 ± 0.000	1.8	1.9	0.5	N/A	N/A	N/A	N/A	N/A	N/A	N/A	N/A
ADG20	0.004 ± 0.001	0.006 ± 0.000	0.010 ± 0.001	0.009 ± 0.000	1.4	2.5	2.2	N/A	N/A	N/A	N/A	N/A	N/A	N/A	N/A
ADG30	0.007 ± 0.002	0.016 ± 0.001	0.029 ± 0.003	0.002 ± 0.001	2.5	4.4	0.3	N/A	N/A	N/A	N/A	N/A	N/A	N/A	N/A
LY-CoV555	0.006 ± 0.002	0.009 ± 0.000	>10	>10	1.5	>1545.3	>1545.3	N/A	N/A	N/A	N/A	N/A	N/A	N/A	N/A
LY-CoV16	0.034 ± 0.007	3.225 ± 1.030	>10	>10	1.5	>291.2	>291.2	N/A	N/A	N/A	N/A	N/A	N/A	N/A	N/A
S309	0.040 ± 0.005	0.078 ± 0.069	0.082 ± 0.002	0.076 ± 0.014	1.5	2.0	1.9	N/A	N/A	N/A	N/A	N/A	N/A	N/A	N/A
222H+222L	0.017 ± 0.001	0.011 ± 0.002	0.016 ± 0.001	0.009 ± 0.000	0.6	1.0	0.5	N/A	N/A	N/A	N/A	N/A	3-53	K	3-20
150H+222L	0.016 ± 0.003	0.010 ± 0.001	0.007 ± 0.001	0.003 ± 0.000	0.6	0.4	0.2	N/A	N/A	N/A	N/A	N/A	3-53	K	3-20
158H+222L	0.033 ± 0.003	0.014 ± 0.001	0.056 ± 0.015	0.019 ± 0.000	0.4	1.7	0.6	N/A	N/A	N/A	N/A	N/A	3-53	K	3-20
175H+222L	>10	0.399 ± 0.012	>10	>10	<0.04	N/A	N/A	N/A	N/A	N/A	N/A	N/A	3-53	K	3-20
269H+222L	0.552 ± 0.085	0.024 ± 0.001	>10	>10	0.04	>18.1	>18.1	N/A	N/A	N/A	N/A	N/A	3-53	K	3-20

Table S3 Results of PDB BLAST for the majority of the mAb 222 light chain sequence, PDB codes shown and residue 30 (within CDR L1) outlined in red. Related to Figure 4.

222	MTQSPGTLSLSPGERATLSCRASQSV-PSSYLAWYQQKPGQAPRLLIYGASTRATGIPDRFSGSGSGTDFTLTISRLEPEDFAVY
7JMO_L	MTQSPGTLSLSPGERATLSCRASQSV-SSSYLAWYQQKPGQAPRLLIYGASSRATGIPDRFSGSGSGTDFTLTISRLEPEDFAVY
4LRN_L	LTQSPGTLSLSPGERATLSCRASQSV-SSSYLAWYQQKPGQAPRLLIYGASSRATGIPDRFSGSGSGTDFTLTISRLEPEDFAVY
7K43_D	MMQSPGTLSLSPGERATLSCRASQSV-SSSYLAWYQQKPGQAPRLLIYGASSRATGIPDRFSGSGSGTDFTLTISRLEPEDFAVY
4OB5_L	LTQSPGTLSLSPGERATLSCRASQSV-SSSYLAWYQQKPGQAPRLLIYGASSRATGIPDRFSGSGSGTDFTLTISRLEPEDFAVY
6OL6_L	LTQSPGTLSLSPGERATLSCRASQSV-SSSYLAWYQQKPGQAPRLLIYGASSRATGIPDRFSGSGSGTDFTLTISRLEPEDFAVY
6O26_B	LTQSPGTLSLSPGERATLSCRASQSV-SSSYLAWYQQKPGQAPRLLIYGASSRATGIPDRFSGSGSGTDFTLTISRLEPEDFAVY
4M62_L	LTQSPGTLSLSPGERATLSCRASQSV-SSSYLAWYQQKPGQAPRLLIYGASSRATGIPDRFSGSGSGTDFTLTISRLEPEDFAVY
5TFS_L	LTQSPGTLSLSPGERATLSCRASQSV-SSSYLAWYQQKPGQAPRLLIYGASSRATGIPDRFSGSGSGTDFTLTISRLEPEDFAVY
6OL5_L	LTQSPGTLSLSPGERATLSCRASQSV-SSSYLAWYQQKPGQAPRLLIYGASSRATGIPDRFSGSGSGTDFTLTISRLEPEDFAVY
5JOF_B	LTQSPGTLSLSPGERATLSCRASQSV-SSSYLAWYQQKPGQAPRLLIYGASSRATGIPDRFSGSGSGTDFTLTISRLEPEDFAVY
7LAB_L	LTQSPGTLSLSPGERATLSCRASQSV-SSSYLAWYQQKPGQAPRLLIYGASSRATGIPDRFSGSGSGTDFTLTISRLEPEDFAVY
6GHG_B	LTQSPGTLSLSPGERATLSCRASQSV-SSSYLAWYQQKPGQAPRLLIYGASSRATGIPDRFSGSGSGTDFTLTISRLEPEDFAVY
6O41_A	LTQSPGTLSLSPGERATLSCRASQSV-SSSYLAWYQQKPGQAPRLLIYGASSRATGIPDRFSGSGSGTDFTLTISRLEPEDFAVY
6WIO_B	LTQSPGTLSLSPGERATLSCRASQSV-SSSYLAWYQQKPGQAPRLLIYGASSRATGIPDRFSGSGSGTDFTLTISRLEPEDFAVY
5I17_A	LTQSPGTLSLSPGERATLSCRASQSV-SSSYLAWYQQKPGQAPRLLIYGASSRATGIPDRFSGSGSGTDFTLTISRLEPEDFAVY
4NP4_L	LTQSPGTLSLSPGERATLSCRASQSV-SSSYLAWYQQKPGQAPRLLIYGASSRATGIPDRFSGSGSGTDFTLTISRLEPEDFAVY
4HWB_L	LTQSPGTLSLSPGERATLSCRASQSI-SSSYLAWYQQKPGQAPRLLIYGASSRATGIPDRFSGSGSGTDFTLTISRLEPEDFAVY
6CA9_A	LTQSPGTLSLSPGERATLSCRASQSV-SSSYLAWYQQKPGQAPRLLIYGASSRATGIPDRFSGSGSGTDFTLTISRLEPEDFAVY
7CDI_L	LTQSPGTLSLSPGERATLSCRASQSV-SSSYLAWYQQKPGQAPRLLIYGASSRATGIPDRFSGSGSGTDFTLTISRLEPEDFAVY
7K8N_B	LTQSPGTLSLSPGERATLSCRASQSV-SSSYLAWYQQKPGQAPRLLIYGASSRATGIPDRFSGSGSGTDFTLTISRLEPEDFAVY
6BKC_L	LTQSPGTLSLSPGERATLSCRASQSV-SSSYLAWYQQKPGQAPRLLIYGASSRATGIPDRFSGSGSGTDFTLTISRLEPEDFAVY
4HWE_L	LTQSPGTLSLSPGERATLSCRASQSI-SSSYLAWYQQKPGQAPRLLIYGASSRATGIPDRFSGSGSGTDFTLTISRLEPEDFAVY
7LK9_B	LTQSPGTLSLSPGERATLSCRASQSV-SSSYLAWYQQKPGQAPRLLIYGASSRATGIPDRFSGSGSGTDFTLTISRLEPEDFAVY
6XE1_L	LTQSPGTLSLSPGERATLSCRASQSV-SSSYLAWYQQKPGQAPRLLIYGASSRATGIPDRFSGSGSGTDFTLTISRLEPEDFAVY
7K8M_B	LTQSPGTLSLSPGERATLSCRASQSV-SSSYLAWYQQKPGQAPRLLIYGASSRATGIPDRFSGSGSGTDFTLTISRLEPEDFAVY
7D0C_F	LTQSPGTLSLSPGERATLSCRASQSV-SSSYLAWYQQKPGQAPRLLIYGASSRATGIPDRFSGSGSGTDFTLTISRLEPEDFAVY
6O25_B	LTQSPGTLSLSPGERATLSCRASQSV-SSSYLAWYQQKPGQAPRLLIYGASSRATGIPDRFSGSGSGTDFTLTISRLEPEDFAVY
5GMQ_C	LTQSPGTLSLSPGERATLSCRASQSV-SSSYLAWYQQKPGQAPRLLIYGASSRATGIPDRFSGSGSGTDFTLTISRLEPEDFAVY
3QEG_L	MMQSPGTLSLSPGERATLSCRASQSV-SSSYLAWYQQKPGQAPRLLIYGASSRATGIPDRFSGSGSGTDFTLTISRLEPEDFAVY
5IBT_L	LTQSPGTLSLSPGERATLSCRASQSV-SSSYLAWYQQKPGQAPRLLIYGASSRATGIPDRFSGSGSGTDFTLTISRLEPEDFAVY
7K45_L	LTQTPGTLSLSPGERATLSCRASQSV-SSSYLAWYQQKPGQAPRLLIYGASSRATGIPDRFSGSGSGTDFTLTISRLEPEDFAVY
6J9O_L	MTQSPGTLSLSPGERATLSCRASQSV-SSSYLAWYQQKPGQAPRLLIYRASSRATGIPDRFSGSGSGTDFTLTISRLEPEDFAVY
6MVL_L	LTQSPGTLSLSPGERATLSCRASQSV-SSSYLAWYQQKPGQAPRLLIYGASSRATGIPDRFSGSGSGTDFTLTISRLEPEDFAVY
1IQD_A	LTQSPGTLSLSPGERATLSCRASQSF-SSSYLAWYQQKPGQAPRLLIYGASTRATGIPDRFSGSGSGTDFTLTISRLEPEDFAVY
5Y2L_J	MTQSPGTLSLSPGERATLSCRASQSV-SSSYLAWYQQKPGQAPRLLIYRASSRATGIPDRFSGSGSGTDFTLTISRLEPEDFAVY
7ND9_L	MTQSPGTLSLSPGERATLSCRASQSV-SSSYLAWYQQKPGQAPRLLIYGASSRATGIPDRFSGSGSGTDFTLTISRLEPEDFGVY
5XMH_C	LTQSPGTLSLSPGERATLSCRASQSI-SSSYLAWYQQRPGQAPRLLIYGASTRATGIPDRFSGSGSGTDFTLTISRLEPEDFAVY
5WKO_B	LTQSPGTLSLSPGERATLSCRASQSV-SSSYLAWYQQKPGQAPRLLIYGASSRATGIPDRFSGSGSGTDFTLTITRLEPEDFAVY
7BEO_D	MTQSPGTLSLSPGERATLSCRASQSV-SSSYLAWYQQKPGQAPRLLIYGASSRATGIPDRFSGSGSGTDFTLTISRLEPEDFGVY
4YPG_A	LTQSPGTLSLSPGERATLSCRASQSV-SSTYLAWYQQKPGQAPRLLIYGASSRATGIPDRFSGSGSGTDFTLTISRLEPEDFAVY
5WI9_E	LTQSPGTLSLSPGERATLSCRASQSV-SSTYLAWYQQKPGQAPRLLIYGASSRATGIPDRFSGSGSGTDFTLTISRLEPEDFAVY
6O28_B	LTQSPGTLSLSPGERATLSCRASQSV-SSTYLAWYQQKPGQAPRLLIYGASSRATGIPDRFSGSGSGTDFTLTISRLEPEDFAVY
7K3Q_L	LTQTPGTLSLSPGERATLSCRASQSV-SSSYLAWYQQKPGQAPRLLIYGASSRATGIPDRFSGSGSGTDFTLTISRLEPEDFAVY
5D1Q_D	LTQSPGTLSLSPGERATLSCRASQSV-SSSYLAWYQQKPGQAPRLLIYGASSRATGIPDRFSGSGSGTDFTLSISRLEPEDFAVY
6CA7_L	LTQSPGTLSLSPGERATLSCRASQSV-SNNYLAWYQQKPGQAPRLLIYGASSRATGIPDRFSGSGSGTDFTLTISRLEPEDFAVY
5K9J_L	LTQSPGTLSLSPGERATLSCRASQSV-ASSYLAWYQQKPGQAPRLLIYGASSRATGVPDRFSGSGSGTDFILTISRLEPEDFAVY
7NDB_L	MTQSPGTLSLSPGERATLSCRASQSV-RSSYLAWYQQKPGQAPRLLIYGASRRGTGIPDRFSGSGSGTDFTLTISRLEPEDFAVY
5K9K_B	LTQSPGTLSLSPGERATLSCRASQSV-ASSYLAWYQQKPGQAPRLLIYGASSRATGVPDRFSGSGSGTDFILTISRLEPEDFAVY
5XJ3_B	LTQSPGTLSLSPGERATLSCRASQSV-GSSYLAWYQQKPGQAPRLLIYGAFSRATGIPDRFSGSGSGTDFTLTISRLEPEDFAVY
5JO4_B	LTQSPGTLSLSPGERATLSCRASQSL-SSKYLAWYQQKPGQAPRLLIYGASSRATGIPDRFSGSGSGTDFTLTISRLEPEDFAVY
6B0A_L	LTQSPGTLSLSPGERATLSCRTSQSV-SSSYLAWYQQKPGQAPRLLMYGASSRATGIPDRFSGSGSGTDFTLTISRLEPEDFAVY
7NDC_E	LTQSPGTLSLSPGERATLSCRASQSI-SGNYLAWYQHKPGQAPRLLIYGASTRATGIPDRFSGSGSGTDFTLTISRLEPEDFAVY
5TRU_L	LTQSPGTLSLSPGERATLSCRASQSV-GSSYLAWYQQKPGQAPRLLIYGAFSRATGIPDRFSGSGSGTDFTLTISRLEPEDFAVY
6WPS_D	LTQSPGTLSLSPGERATLSCRASQTV-SSTSLAWYQQKPGQAPRLLIYGASSRATGIPDRFSGSGSGTDFTLTISRLEPEDFAVY
6EHV_X	LTQSPGTLSLSPGERATLSCRASQTV-SGSYLAWYQQKPGQAPRLLIYGASTRATGIPDRFSGSGSGTDFTLTISRLEPEDFAVY
6B3M_D	LTQSPGTLSLSPGERATLSCRASQSV-SSNYLAWFQQKPGQAPRLLIYGASNRATGIPDRFSGSGSGTDFTLAISRLEPEDFAVY
5W1K_C	LTQSPGTLSLSPGETATLSCRASQSV-SSSYLAWYQQKPGQAPRLLIYGASGRATGIPDRFSGSGSGTDFTLTISRLEPEDFAVY
6XC4_L	LTQSPGTLSLSPGERATLSCRASQSV-SS-YLAWYQQKPGQAPRLLIYGASSRATGIPDRFSGSGSGTDFTLTISRLEPEDFAVY
6CMG_B	MTQSPGTPSLSPGERATLSCRASQSI-RSTYLAWYQQKPGQAPRLLIYGASSRATGIPDRFSGSGSGTDFTLTISRLEPEDFAVY
7KN6_L	LTQSPGTLSLSPGERATLSCRASQSV-SS-YLAWYQQKPGQAPRLLIYGASSRATGIPDRFSGSGSGTDFTLTISRLEPEDFAVY
4FQL_L	LTQSPGTLSLSPGERATLSCRASQSV-SSSYLAWYQQKPGQAPRLLIYGASTRATGIPARFSGSGSGTDFTLTISRLEPEDLAVY
5X8M_C	LTQSPGTLSLSPGERATLSCRASQRV-SSSYLAWYQQKPGQAPRLLIYDASSRATGIPDRFSGSGSGTDFTLTISRLEPEDFAVY
5XJ4_L	LTQSPGTLSLSPGERATLSCRASQRV-SSSYLAWYQQKPGQAPRLLIYDASSRATGIPDRFSGSGSGTDFTLTISRLEPEDFAVY
5Y0A_E	LTQSPGTLSLSPGERATLSCRASQSV-SSSYLAWYQQKRGQAPRLLIYDASSRATGIPDRFSGSGSGTDFTLTISRLEPEDFAVY
1U6A_L	LTQSPGTLSLSAGERATLSCRASQSV-SSRYLAWYQQKPGQAPRLLIYGASSRATGIPDRFSGSGSGTDFTLTISRVEPEDFAVY
6WS6_B	LTQSPGTLSLSPGERATLSCRASQTV-SSTSLAWYQQKPGQAPRLLIYGASSRATGIPDRFSGSGSGTDFTLTISRLEPEDFAVY
6B9J_B	LTQSPGTLSLSPGERATLSCRASQSV-TSTYLAWHQQKPGQAPRLLIYSASSRATGIPDRFSGSGSGTDFTLTISRLEPEDFAVY
4KXZ_I	LTQSPGTLSLSPGERATLSCRASQSL-GSSYLAWYQQKPGQAPRLLIYGASSRAPGIPDRFSGSGSGTDFTLTISRLEPEDFAVY
7BEN_F	MTQSPGTLSLSPGEGATLSCRASQSV-SSSYLAWYQQKPGQAPRLLIYGASSGATGIPDRFSGSGSGTDFTLTISRLEPEDFAVY
7K8U_L	LTQSPGTVSLSPGERATLSCWASQSV-SASYLAWYQQKPGQAPRLLIYGASSRATGIPDRFSGSGSGTDFTLTISRLEPEDFAVY
3SO3_B	LTQSPGTLSLSPGERATLSCRASQSV-SSSYLAWYQQKPGQAPRLLIYGASTRATGIPARFSGSGSGTDFTLTINSLEPEDFAVY
4QXG_L	LTQSPGTLSLSPGERATLSCRASQSV-SSSFFAWYQQKPGQAPRLLIYGASSRATGIPDRLSGSGSGTDFTLTITRLEPEDFAVY
6BKB_L	LTQSPGTLSLSPGKRATLSCRASQSV-SGSYLAWYQQKPGQAPRLLIYGASNRATGIPHRFSGSGSGTDFTLTISRLEPEDFAVY
5D1X_D	LTQTPATLSLTPGERATLTCRASQSV-SSSYLAWYQQKPGQAPRLLIYGASSRATGIPDRFSGSGSGTDFTLSISRLEPEDFAVY
3W9D_B	LTQSPGTLSLSPGERATLSCRASQSV-TSSQLAWYQQKPGQAPRLLISGASNRATGIPDRFSGSGSGTDFTLTISRLEPEDFAVY
4KV5_E	LTQSPGTLSLSPGERATLSCRASQSL-GSSYLAWYQQKPGQAPRLLIYGASSRAPGIPDRFSGSGSGTDFTLTISRLEPEDFAVY
4IDJ_L	LTQSPGTLSLSPGERATLSCRASQTI-SNNFVAWYQQKPGQAPRLLIYGASTRATGIPDRFSGSGSGTDFTLTISSLEPEDFAVY
4N90_E	LTQSPGTLSLSPGERATLSCRASQGI-SRSYLAWYQQKPGQAPSLLIYGASSRATGIPDRFSGSGSGTDFTLTISRLEPEDFAVY
6WO4_L	LTQSPGTLSLSPGERATLSCRASHSV-SSSNLAWYQQKPGQAPRLLMYGASRRATGIPDRFSGSGSGTDFTLTISRLEPEDFAVY
6OZ9_L	LTQSPGTLSLSPGERATLSCRASQSV-SSSYFAWYQQKPGQAPRLLISGTSTRAPGIPDRFSGSGSGTDFTLTISRLEPEDFAVY
5WYM_A	LTQSPATLSLSPGERATLSCRASQSI-S-SYLAWYQQKPGQAPRLLIYGASTRATGIPDRFSGSGSGTDFTLTISRLEPEDFAVY
7JOO_L	LTQSPGTLSLSPGERATLSCRASQSV-SRSYLAWYQQKRGQAPRLLIYGASSRATGIPDRFSGDGSGTDFTLSISRLEPEDFAVY
5V7R_L	MTQSPDTLSLSPGERATLSCRASQII-SSNYLAWYQQQPGQAPRLLIYGASSRATGIPDRFSGSGSATDFTLTISRLEPEDFAVY
7AH1_A	LTQSPGTLSLSPGERATLSCRASQSVSS-SFLAWYQQKPGQAPRLLIYYASSRATGIPDRFSGSGSGTDFTLTISRLEPEDFAVY
3SDY_L	MTQSPGTLSLSPGERATLSCRASQSV-SMNYLAWFQQKPGQAPRLLIYGASRRATGIPDRISGSGSGTDFTLTISRLEPADFAVY
6N7J_B	MTQSPGTLSLSPGERATLSCRASQSV-PRNYIGWFQQKPGQAPRLLIYGASSRAAGFPDRFSGSGSGTDFTLTITRLEPEDFAMY
1DN0_A	LTQSPATLSLSPGERATLSCGASQSV-SSNYLAWYQQKPGQAPRLLIYDASSRATGIPDRFSGSGSGTDFTLTISRLEPEDFAVY
5UJZ_G	LTQSPGTLSLSPGERATLSCRASQSISG-YYLTWYQQKPGQAPRLLIYGASSRATGIPDRFSGSGSGTDFTLTISRLEPEDFAVY
6MTQ_L	LTQSPDTLSLSPGERATLSCRASQSV-GSSYFAWYQQKPGQAPRLLIYGASTRATGIPGRFSGSGSGTDFTLAITRVEPEDFAVY
6GFE_L	LTQSPATLSLSPGERATLSCRASQSV-RSNYLAWYQQKPGQAPRLLIYGASNRATGVPARFSGSGSGTDFTLTISSLEPEDFAVY
7CH5_L	LTQSPGTLSLSPGERATLSCRASQGV-SS-FLAWYQQKPGQAPRLLIHGASSRATGIPDRFSGSGSGTDFTLTITRLEPEDFAVY
5CSZ_B	LTQSPATLSLSPGERATLSCRASQSV-SSSYLAWYQQKPGQAPRLLIYGASSRATGVPARFSGSGSGTDFTLTISSLEPEDFATY
6BKD_L	LTQSPGTLSLSPGERATLSCRAGQTV-ASNSLAWYQHKPGQAPRLLIYGASIRASGIPDRFSGSGSGTDFTLTISRLEPEDFAVY
5IBU_B	LTQSPGTLSLSPGERATLSCRASQSV-FSTFLAWYQQKPGQAPRLLIYAASRRAAGIPDRFSGSESGTDFTLTISRLEPEDFAVY
1RHH_A	LTQSPGTLSLSAGERATLSCRASQSV-SSGSLAWYQQKPGQAPRLLIYGASTRATGIPDRFSGSGSGTDFTLTIGRLEPEDLAVY
6WIY_L	LTQSPGTLSLSPGERVTLSCRASQTV-YNSYLAWYQQKPGQAPTLLIYGTSTRATGVPDRFSGSGSGTVFTLTISRLEPEDFAVY
6WIZ_L	LTQSPGTLSLSPGERVTLSCRASQTV-YNSYLAWYQQKPGQAPTLLIYGTSTRATGVPDRFSGSGSGTVFTLTISRLEPEDFAVY
5GS1_A	LTQSPATLSLSPGERATLSCRASQSV-SSNYLAWYQQKPGQAPRLLIYDSSSRATGVPARFSGSGSGTDFTLTISSLEPEDFAVY
5W4L_C	LTQSPGTLSMSPGERATLSCRASRTV-SSSNLAWYQQKPGQAPRLLIYDVSSRATGIPDRFSGRGSGTDFTLTISRLEPEDFAVY

Table S4A Neutralization of Victoria and P.1 virus by convalescent plasma (infection with original Victoria-like virus), related to Figure 6

Convalescent plasma	FRNT50 (Recprocal plasma dilution)		Victoria/P.1 ratio
	Victoria	P.1	
Convalescent 1	61	25	2.5
Convalescent 2	689	49	14.2
Convalescent 3	526	173	3.0
Convalescent 4	409	356	1.1
Convalescent 5	369	105	3.5
Convalescent 6	1270	556	2.3
Convalescent 7	274	80	3.4
Convalescent 8	633	223	2.8
Convalescent 9	667	126	5.3
Convalescent 10	124	34	3.7
Convalescent 11	102	30	3.4
Convalescent 12	339	74	4.6
Convalescent 13	331	240	1.4
Convalescent 14	438	95	4.6
Convalescent 15	6397	3261	2.0
Convalescent 16	44	39	1.1
Convalescent 17	1115	87	12.8
Convalescent 18	242	64	3.8
Convalescent 19	29	20	1.4
Convalescent 20	154	136	1.1
Convalescent 21	487	165	3.0
Convalescent 22	438	241	1.8
Convalescent 23	381	83	4.6
Convalescent 24	1647	390	4.2
Convalescent 25	913	322	2.8
Convalescent 26	1880	825	2.3
Convalescent 27	1464	206	7.1
Convalescent 28	361	81	4.4
Convalescent 29	2859	1010	2.8
Convalescent 30	1109	477	2.3
Convalescent 31	811	274	3.0
Convalescent 32	395	130	3.0
Convalescent 33	1144	207	5.5
Convalescent 34	676	201	3.4

Table S4B Neutralization of Victoria and P.1 virus by B.1.1.7 plasma, related to Figure 6

B.1.1.7 plasma	Day post symptom onset	FRNT50 (Recprocal plasma dilution)		Victoria/P.1 ratio
		Victoria	P.1	
B.1.1.7 P3	11	440	116	3.8
B.1.1.7 P4	18	136884	48440	2.8
B.1.1.7 P5	45	1506	787	1.9
B.1.1.7 P6	31	370	259	1.4
B.1.1.7 P7	33	2250	267	8.4
B.1.1.7 P8	25	2999	2261	1.3
B.1.1.7 P9	26	970	861	1.1
B.1.1.7 P10	18	3735	861	4.3
B.1.1.7 P11	24	2193	1116	2.0
B.1.1.7 P13	29	<20	67	<0.3
B.1.1.7 P14	21	1700	168	10.1
B.1.1.7 P15	16	168	414	0.4

Table S5 Neutralisation of Victoria and P.1 viruses by immune serum from vaccinees, related to Figure 7

Vaccine samples	Day Post-boost	FRNT50 (Recprocal serum dilution)		Victoria/P.1 ratio
		Victoria	P.1	
Pfizer1	7	1149	396	2.9
Pfizer2	7	<20	36	<0.6
Pfizer3	7	1727	698	2.5
Pfizer4	8	2234	712	3.1
Pfizer5	7	3016	1033	2.9
Pfizer6	7	1521	302	5.0
Pfizer7	7	609	294	2.1
Pfizer8	7	4340	2119	2.0
Pfizer9	7	1467	361	4.1
Pfizer10	7	1757	343	5.1
Pfizer11	7	860	424	2.0
Pfizer12	7	1749	452	3.9
Pfizer13	7	1851	669	2.8
Pfizer14	7	407	294	1.4
Pfizer15	8	1285	571	2.3
Pfizer16	8	1286	311	4.1
Pfizer17	8	1810	304	6.0
Pfizer18	8	1198	282	4.3
Pfizer19	8	466	229	2.0
Pfizer20	8	1539	693	2.2
Pfizer21	9	184	52	3.5
Pfizer22	11	1061	491	2.2
Pfizer23	12	1658	355	4.7
Pfizer24	12	1155	569	2.0
Pfizer25	15	8092	5029	1.6
AstraZeneca 1	28	495	265	1.9
AstraZeneca 2	28	580	429	1.4
AstraZeneca 3	28	253	<20	>12.6
AstraZeneca 4	28	183	102	1.8
AstraZeneca 5	28	432	215	2.0
AstraZeneca 6	28	764	111	6.9
AstraZeneca 7	28	133	29	4.5
AstraZeneca 8	28	257	116	2.2
AstraZeneca 9	28	501	97	5.2
AstraZeneca 10	28	357	133	2.7
AstraZeneca 11	14	334	115	2.9
AstraZeneca 12	14	250	94	2.7
AstraZeneca 13	14	122	40	3.1
AstraZeneca 14	14	212	110	1.9
AstraZeneca 15	14	789	281	2.8
AstraZeneca 16	14	538	181	3.0
AstraZeneca 17	14	1159	359	3.2
AstraZeneca 18	14	353	85	4.1
AstraZeneca 19	14	975	382	2.6
AstraZeneca 20	14	169	74	2.3
AstraZeneca 21	14	155	87	1.8
AstraZeneca 22	14	152	98	1.5
AstraZeneca 23	14	126	67	1.9
AstraZeneca 24	14	293	151	1.9
AstraZeneca 25	14	94	25	3.8
